# Supplementary material for: Quick and Easy Covalent Grafting of Sulfonated Dyes to CMC: From Synthesis to Colorimetric Sensing Applications
Source: Polymers (Basel). 2022 Sep 27;14(19):4061. doi: 10.3390/polym14194061 (PMC9571709; doi:10.3390/polym14194061)
Supplement: Supplementary file 1 [file polymers-14-04061-s001.zip › polymers-1932108-supplementary.pdf]

polymers

MDPI

Supplementary Materials: Quick and Easy Covalent Grafting of Sulfonated Dyes to CMC: From Synthesis to Colorimetric Sensing Applications

Lisa Rita Magnaghi, Camilla Zanoni, Denise Bellotti, Giancarla Alberti, Paolo Quadrelli and Raffaela Biesuz

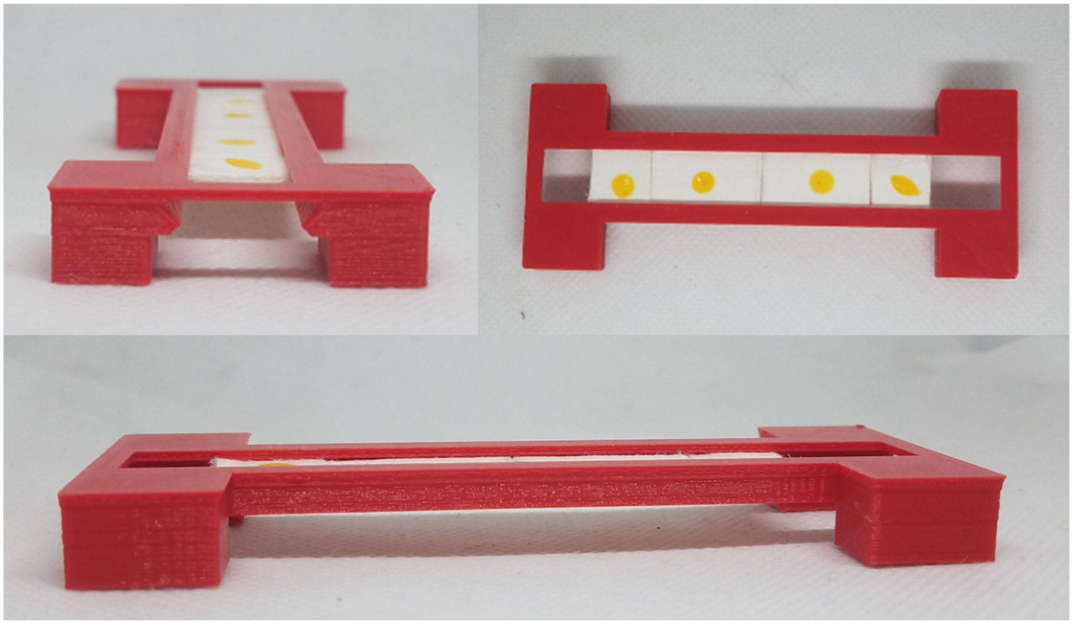

Figure S1. 3D-printed support for sensors exposure over vapour-generating solutions or solid samples.

S1. Drop casting deposition optimisation: Experiments list for the 2^3 full factorial design.

Table S1. Experimental plan for drop casting deposition optimisation and NaOH addition required for equilibration at alkaline pH.

n	CR-CMC@ concentration (% w/v H_2O)	Glycerol/CR-CMC@ ratio	Drop volume (µL)	Equilibration to alkaline pH
1	7.5 %	1	20 µL	10 µL NaOH 0.075M
2	5 %	1	20 µL	10 µL NaOH 0.1M
3	7.5 %	0.5	20 µL	10 µL NaOH 0.075M
4	5 %	0.5	20 µL	10 µL NaOH 0.05M
5	7.5 %	1	10 µL	20 µL NaOH 0.1M
6	5 %	1	10 µL	15 µL NaOH 0.05M
7	7.5 %	0.5	10 µL	20 µL NaOH 0.1M
8	5%	0.5	10 µL	15 µL NaOH 0.1M
9	6.25 %	0.75	15 µL	15 L NaOH 0.1M

S2. General procedure for picture acquisition and analysis

Pictures of the sensors were taken by a NIKON COOLPIX S6200 portable camera equipped with a 1/2.3″ (6.16 mm 4.62 mm, crop factor 5.6) 16 mpx CCD sensor. A portable led lightbox (23 cm x 23 cm x 23 cm), equipped with 20 LEDs (550LM, colour temperature 5500 K) was used to guarantee the reproducibility of the photos (PULUZ, Photography

Light Box, Shenzhen Puluz Technology Limited, Shenzhen, China). Setting ISO at the lowest possible for the camera (80) and using the lightbox, all the pictures were acquired at shutter speed 1/60 s and aperture f/3.2. The white balance was kept constant for all the images by setting a white reference point inside the lightbox. The pictures (4608 x 3456 pixels) were acquired as a .jpg file using a neutral photo profile from the camera. GIMP software was used to acquire HSL values from the .jpg files straight from the camera, manually selecting the region of interest (ROI) by exploiting the "Intelligent Scissors" tool.

S3. Physico-chemical characterisation of CR-CMC@

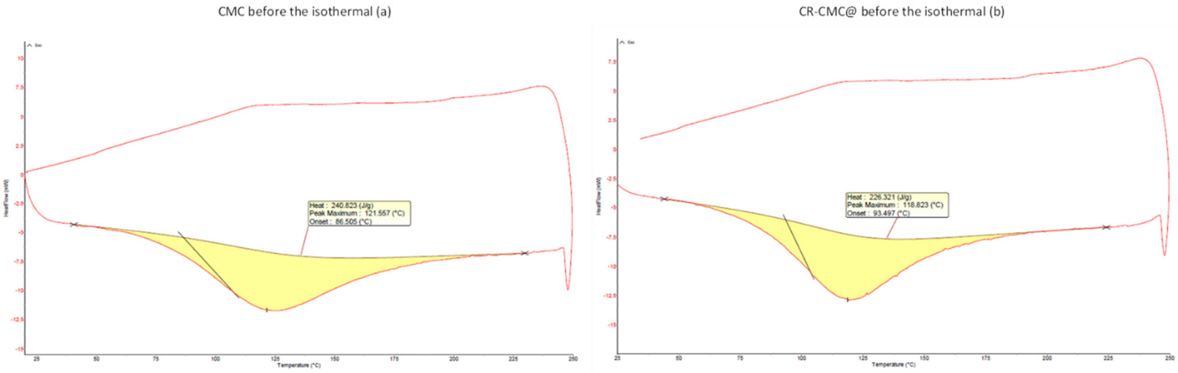

Figure S2. Calorimetric profiles for CMC (a) and CR-CMC@ (b) before the isothermal.

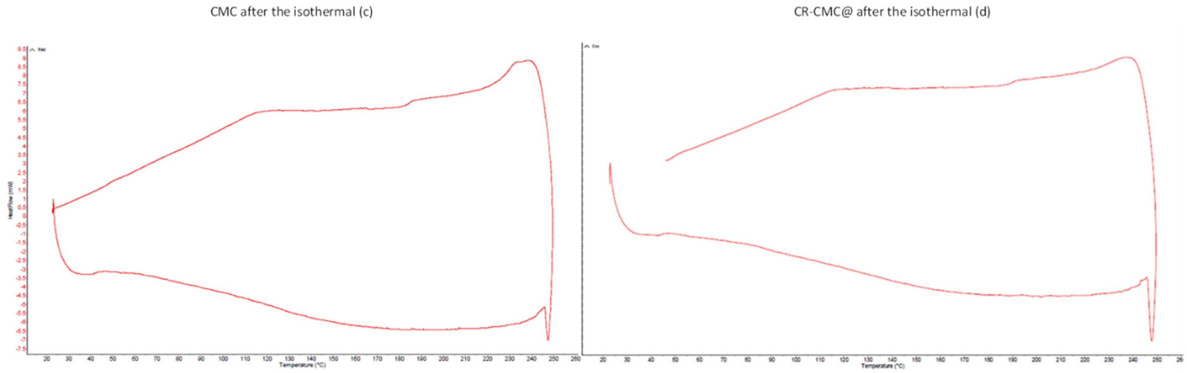

Figure S3. Calorimetric profiles for CMC (a) and CR-CMC@ (b) after the isothermal.

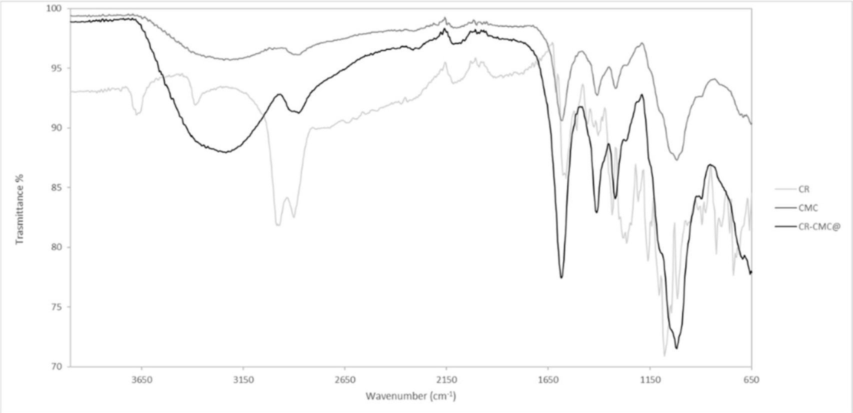

Figure S4. FT-IR spectra of CR (light grey), CMC (grey) and CR-CMC@ (black).

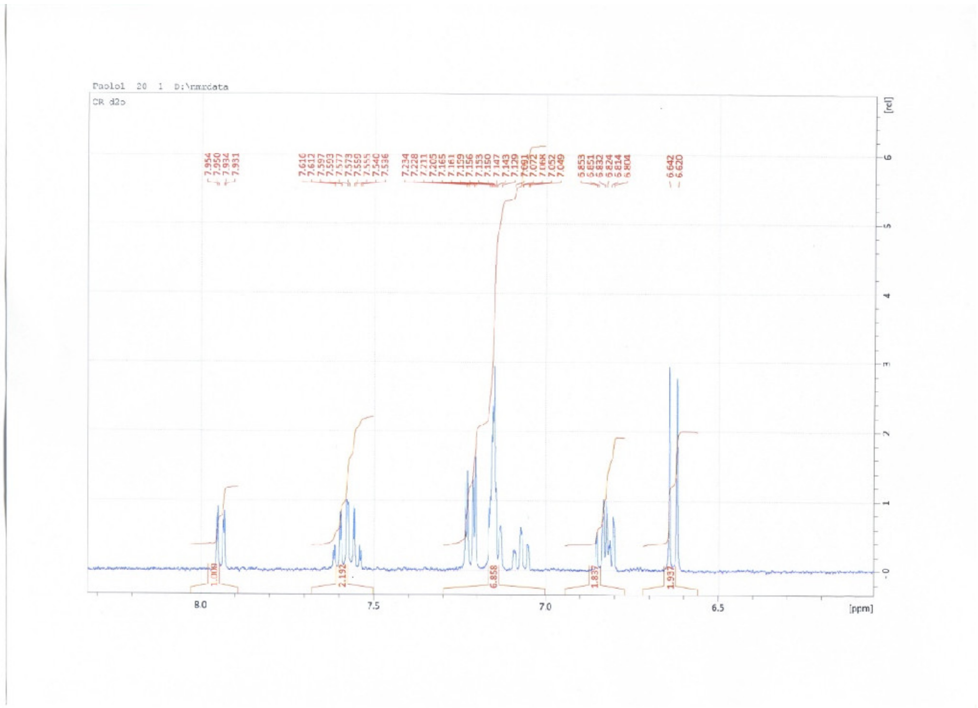

Figure S5. ^{1}H-NMR of CR in D_2O (zoom on the aromatic region).

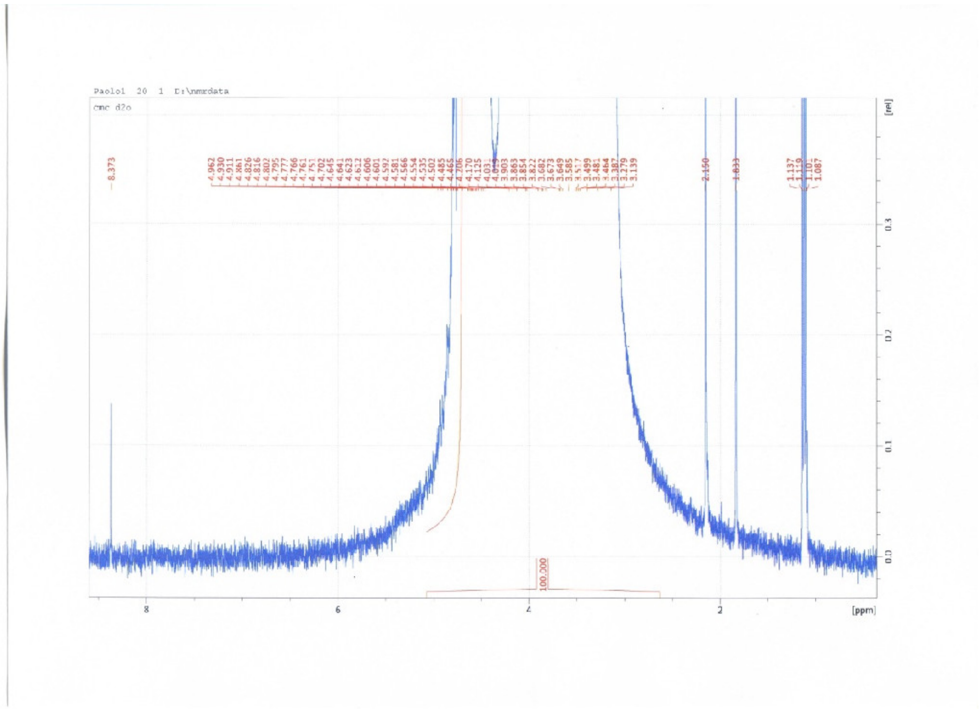

Figure S6. ^{1}H-NMR of CMC in D_2O (zoom on the aromatic region).

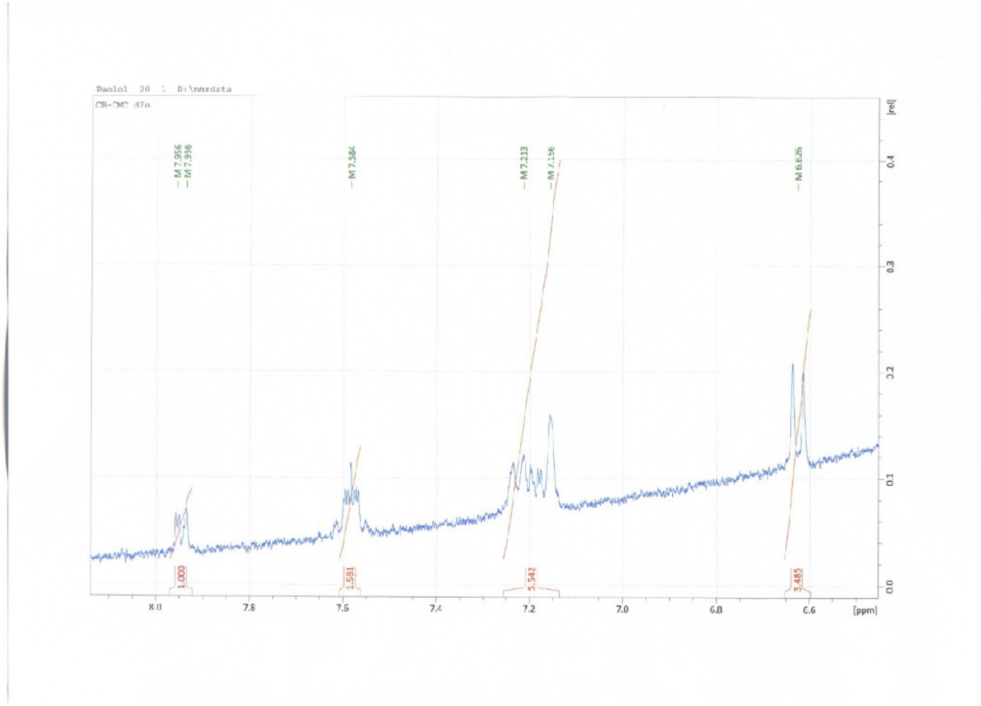

Figure S7. ^{1}H-NMR of CR-CMC@ in D_2O (zoom on the aromatic region).

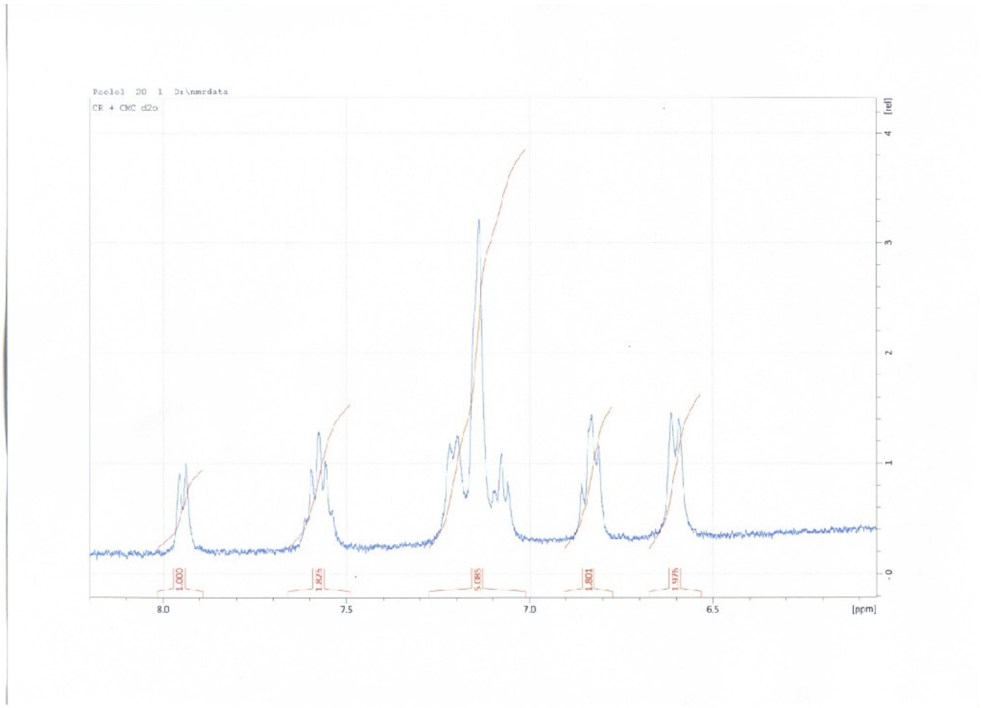

Figure S8. ^{1}H-NMR of CR and CMC mixture in D_2O (zoom on the aromatic region).

S4.Protonation equilibria of CR, CMC and CR-CMC@

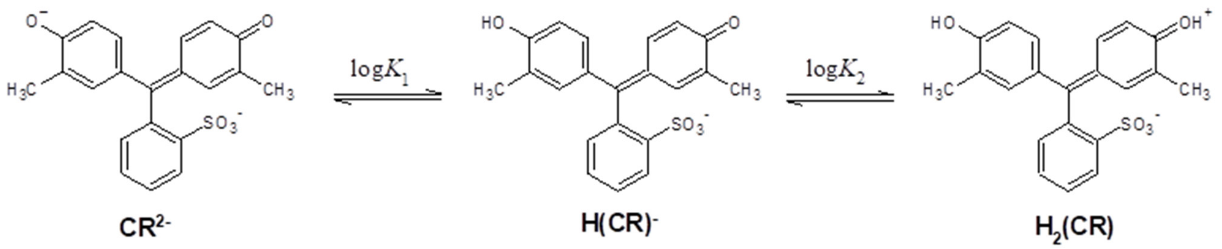

Figure S9. Cresol red (CR) protonation equilibria.

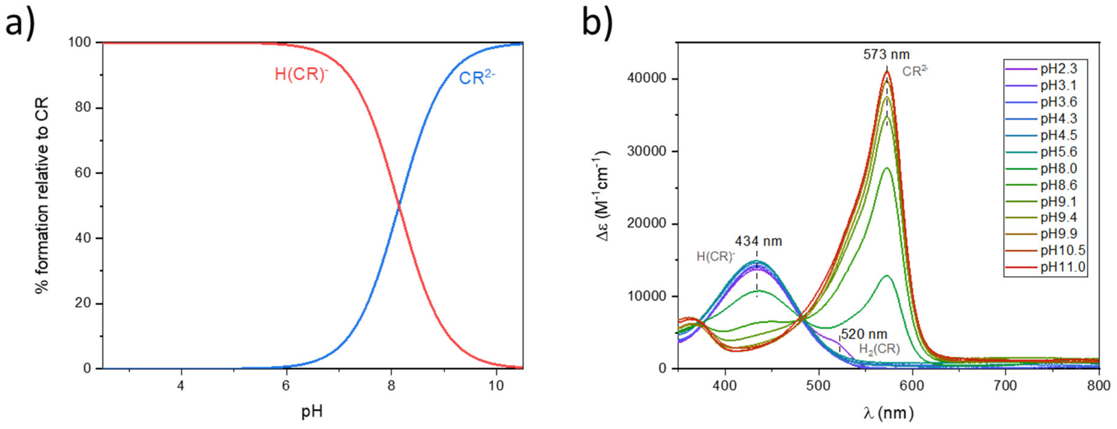

Figure S10. Species distribution diagram for protonation equilibria of CR in the explored pH range (a) and UV-Vis absorption spectra of CR at different pH values. CCR = 1×10^{-5} M, optical path 1 cm. The wavelengths of maximum absorption of the three protonated forms of CR are also reported.

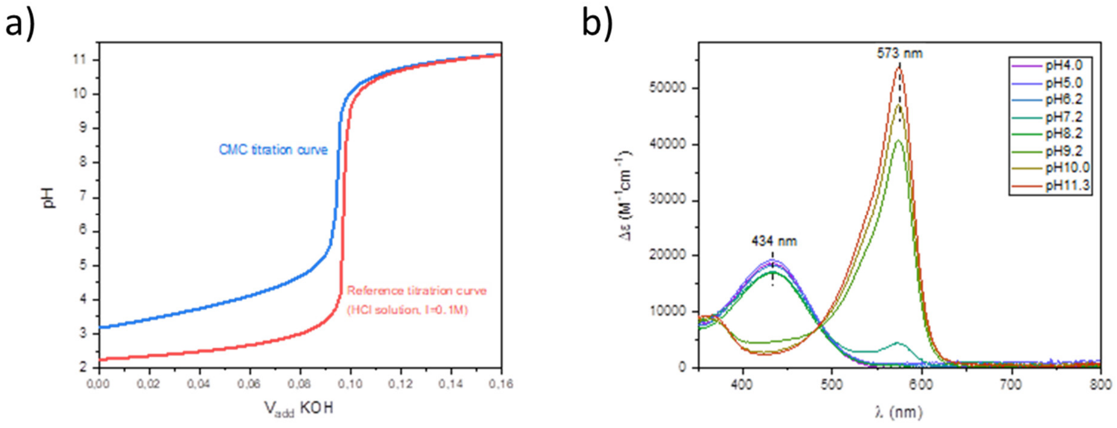

Figure S11. Titration curves of an aqueous solution in the presence and absence of dissolved CMC (C_{CMC}=0.25% w/v) (a) and UV-Vis absorption spectra of CR-CMC@ (0.25% w/V) at different pH values. C_{CR} = 4.8 × 10-6 M, optical path 1 cm. The wavelengths of maximum absorption of the three prot-nated forms of CR are also reported.

S5.Evolution of H and S values in the HSL space color during detection of volatile AcOH

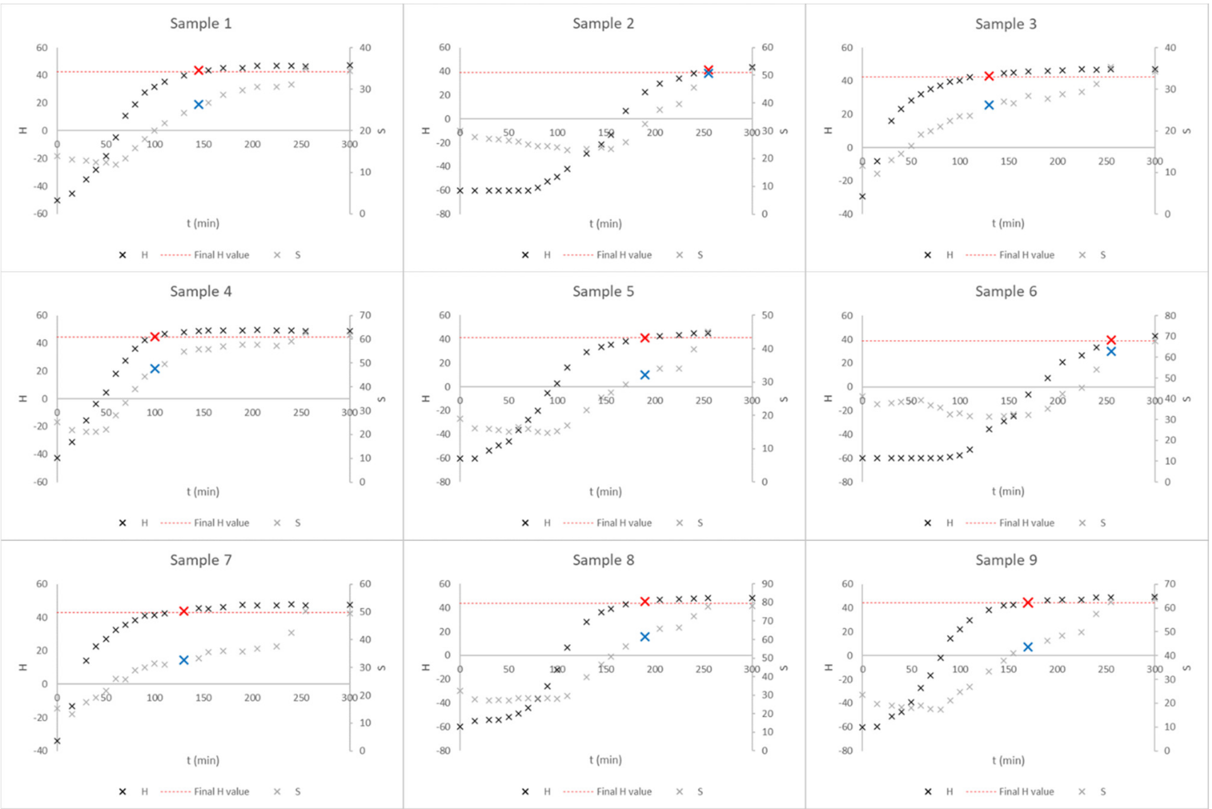

Figure S12. Evolution of H (black) and S (grey) registered for samples from 1 to 9 during exposure to vapors originated from 100 mL AcOH 0.01M in a sealed box (V=1.75 L). The red dashed line in each plot represents the final H value reduced by 10%, which means when the detection is labelled as complete; the red and blue x highlighted H and S value when this threshold is crossed.

S6.Drop casting deposition optimisation: model equations

Once run the experiments and collected the responses value for conversion time (R1) and color intensity (R2), the coefficients were calculated for both the responses, as reported in Figure S5. As for R1, the highest effect was associated with x2, i.e. glycerol/CR-CMC@ ratio, while x1 (CR-CMC@ concentration) and x3 (drop volume) presented a lower effect. Both x1 and x3 had a positive effect meaning that the response was increased for higher values of both the variables or, opposite, was decreased decreasing both CR-CMC@ concentration and drop volume, as we could expect. On the other hand, x2 presented a negative effect suggesting that the conversion time was decreased for higher glycerol content and increased for lower contents.

Moving to R2, the highest effect was mainly associated with x1, being CR-CMC@ the only coloured compound in the aqueous mixture, and also to x3, even if with a lower effect. Therefore, color intensity could be increased at higher values of CR-CMC@ concentrations and for bigger drops.

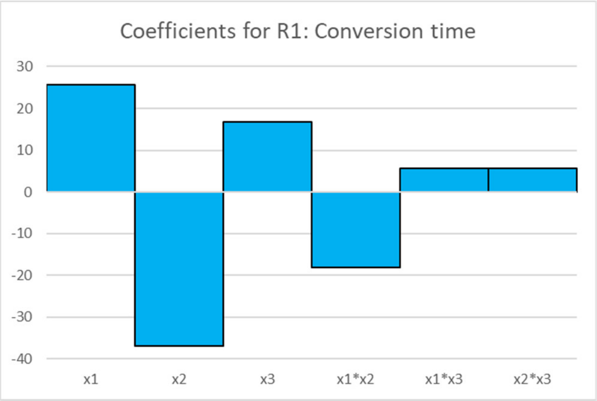

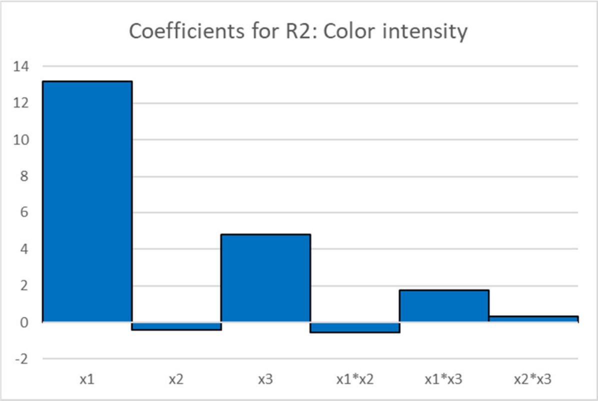

Figure S13. Coefficients calculated for R1, conversion time, on the left and R2, color intensity, on the right.

Finally, to evaluate model reliability and validate it, the comparison between experimental and fitted responses value was performed, both for the sensors exploited to build the models, labelled from 1 to 8 in Table S1, and three replicates of the centre point, described as sample 9 in Table 1S. As we could clearly observe from the graphs reported in Figure S6, the fitted values showed a better agreement with the experimental ones in the case of R2 but, in both cases, the models were validated since the centre point overlapped the y=x line, taking into account its experimental standard deviation, reported in bars.

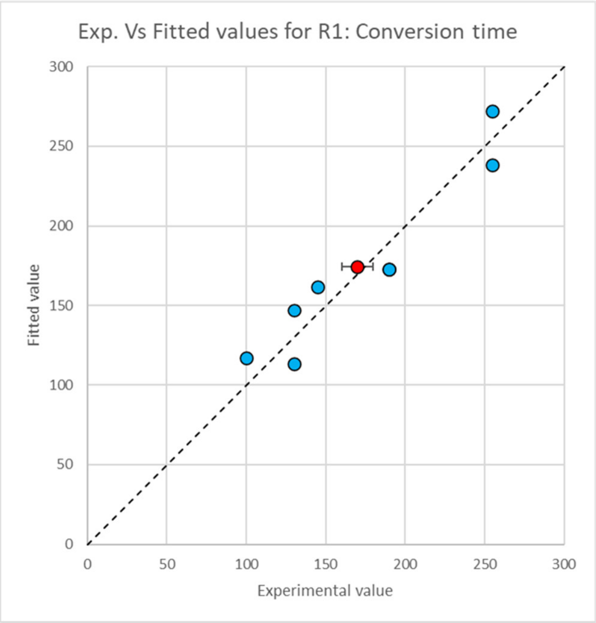

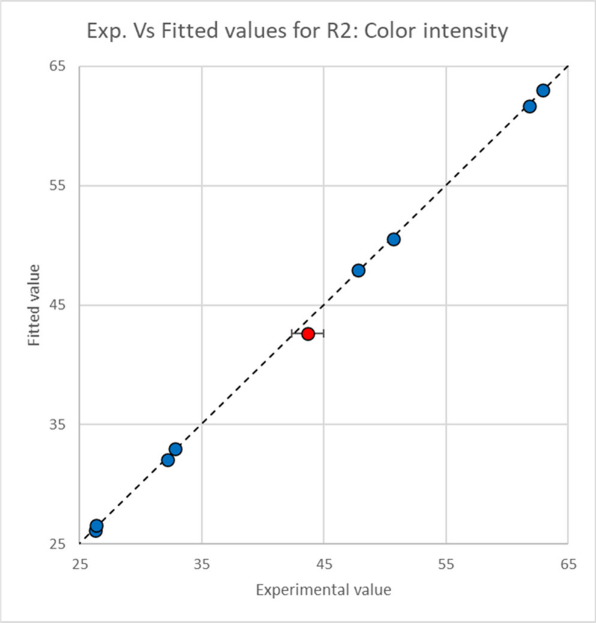

Figure S14. Experimental vs. Fitted values for R1, conversion time, on the left and R2, color intensity, on the right for training samples (blue) and test point (red).
